# Tobacco Induced Diseases: A World-Wide Concern

**DOI:** 10.1186/1617-9625-1-14

**Published:** 2003-09-15

## 

Prior to September 11, 2001, I along with many other Americans viewed the potential of terrorist attacks as something that occurred "over there" and "not here." On that day, we began to understand that we are all part of a global community where events in one part of the world impact so many others. And, that we – all citizens of the world – share problems and plagues, as well as the potential for solutions and health. Our differences are, perhaps, outweighed by our similarities. Clearly, the impact of tobacco induced disease has been an equal opportunity visitor to all countries of the globe. The current issue of this journal reflects that global concern.

In this issue of the journal there are seven excellent contributions to the global task of addressing the problem of tobacco addiction and its health consequences. They are from all corners of the world – Canada (two contributions), Sweden, the United Kingdom, Germany, the United States and Japan – and are representative of the Society for the Prevention of Tobacco Induced Diseases (PTID) membership. They also illustrate the broad range of interest, topics, and research methods needed to examine the issue of tobacco addiction, with studies ranging from behavioral interventions to basic science examinations.

Drs. Chan and Koren from Canada address the many concerns associated with smoking during pregnancy, including miscarriage, preterm delivery and stillbirth. In this extensive review of published studies – 122 are reviewed – the results reveal greater nicotine metabolism in pregnant individuals who continue to smoke during pregnancy. The studies support current guidelines from several health organizations, which uniformly recommend that Nicotine Replacement Therapy should be considered if non-pharmacological therapies have been unsuccessful. The authors conclude with a call for further studies on the safety and effectiveness of Nicotine Replacement Therapy and bupropion use during pregnancy. However, they stress that given the current research and guidelines, pharmacological cessation aids should be considered if non-pharmacological therapies have not been effective.

Drs. Johansson, Halling, and the LinQuest Study Group from Sweden point out that smoking prevalence and smoking behaviours have changed in society, and an increased awareness of the importance of protecting children from environmental tobacco smoke (ETS) is reported. Their study aims to determine if smoking prevalence and smoking behaviours were influenced by parenthood, and if differences in health-related quality of life differed between smoking and non-smoking parents. The researchers find that parenthood did not seem to be associated with lower smoking prevalence. Logistic regression models showed that smoking prevalence was significantly associated with education, gender and mental health. Smoking behaviour, as well as attitudes toward passive smoking, seemed to be influenced by parenthood. Johansson and colleagues conclude that smoking behaviour, but not smoking prevalence, seems to be influenced by parenthood, and they stress the importance of commonly-used precautions when children's risk for ETS exposure is estimated.

Drs. Unal, Critchley, and Capewell from the United Kingdom explore how much of the coronary heart disease (CHD) mortality fall in England and Wales can be attributed to changes in smoking prevalence. Their study uses a previously validated cell-based IMPACT CHD mortality model to estimate the deaths prevented or postponed by changes in population smoking prevalence in England and Wales between 1981 and 2000. They report that large declines in smoking prevalence accounted for 29,460 fewer CHD deaths in England and Wales in 2000 compared with 1981. This emphasizes the importance of employing a national strategy with comprehensive tobacco control programs to further reduce smoking. It would be prudent for those in other countries to consider these recommendations as well.

There are four basic science contributions in this issue. Dr. Meisel and colleagues from Germany address the significant dose-effect relationship between the exposure to tobacco smoke and the extent of periodontal disease, assessed as attachment loss and tooth loss. They provide support for the hypothesis that subjects who smoke and who bear genetic variants of polymorphically expressed phenotypes are at an increased risk of periodontitis. This may occur through the influence of smoking-related impairment on defense mechanisms, rather than on the pathogenic pathways.

Drs. Lam and colleagues from Canada demonstrate the direct in vitro release of the free radical nitric oxide (^•^NO) from extracts and components of smokeless tobacco, in phosphate buffered saline and human saliva using electron spin resonance and chemiluminescence detection. Their findings suggest that tobacco xenobiotics represent as yet unrecognized sources of ^•^NO in the body.

Dr. Chowdhury from the United States addresses the critical issue of cigarette smoking as a known major risk factor for pancreatic cancer and pancreatitis. He systematically studied the pathogenesis of pancreatitis and pancreatic cancer using a rodent model. His results suggest that alterations in metabolic, hormonal and pathologic parameters following nicotine treatment appear consistent with diagnostic criteria of human pancreatitis. Thus, it is likely that rats could be considered as a potential animal model to study the pathogenesis of pancreatitis.

Finally Drs. Aoshiba and Nagai from Japan review and discuss oxidative stress, cell death, and other damage to alveolar epithelial cells as induced by cigarette smoke.

These excellent articles provide the reader with an opportunity to explore issues that arise during the global task of improving the world health. We welcome letters to the editor regarding these contributions as yet another method to further our knowledge of these important lines of inquiry.

Daniel R. Longo, ScD

Editor

Columbia, Missouri, US

## Competing interests

The authors declare that they have no competing interests.

